# How Do Cells Make Decisions: Engineering Micro- and Nanoenvironments for Cell Migration

**DOI:** 10.1155/2010/363106

**Published:** 2010-06-23

**Authors:** Siti Hawa Ngalim, Astrid Magenau, Guillaume Le Saux, J. Justin Gooding, Katharina Gaus

**Affiliations:** ^1^Centre for Vascular Research, University of New South Wales, Sydney 2052 NSW, Australia; ^2^School of Chemistry, University of New South Wales, Sydney 2052 NSW, Australia

## Abstract

Cell migration contributes to cancer metastasis and involves cell adhesion to the extracellular matrix (ECM), force generation through the cell's cytoskeletal, and finally cell detachment. Both adhesive cues from the ECM and soluble cues from neighbouring cells and tissue trigger intracellular signalling pathways that are essential for cell migration. While the machinery of many signalling pathways is relatively well understood, how hierarchies of different and conflicting signals are established is a new area of cellular cancer research. We examine the recent advances in microfabrication, microfluidics, and nanotechnology that can be utilized to engineer micro- and nanoscaled cellular environments. Controlling both adhesive and soluble cues for migration may allow us to decipher how cells become motile, choose the direction for migration, and how oncogenic transformations influences these decision-making processes.

## 1. Introduction

Cell migration is an essential element in development, tissue repair and immune surveillance but can become aberrant in cancer leading to malignant invasion of local tissues and metastasis in distant organs. Cell migration is a complex process that integrates numerous extracellular stimuli but also provides feedback to the microenvironment, which can modulate the structure and chemical signature of the extracellular matrix (ECM). Extracellular stimuli can be chemical cues released from cells of the same or different cell type; adhesive cues in the form of cellular ligands that are part of or attached to the ECM or neighbouring cells as well as mechanical forces and structural features within tissue and the ECM. All these parameters contribute to the initiation of migration from a quiescent state, direction and speed of migration and interactions with bystander cells and ECM components.

One can distinguish between different modes of cell migration with mesenchymal and amoeboid cell migrations being two modes of single cell migration. Mesenchymal cell migration, as seen in fibroblast, is characterized by an elongated morphology and firm adhesion to the microenvironment. Amoeboid cell migration such as that of lymphocytes, has dynamic focal complexes and high degree of deformability [1]. Collective cell migration has a similar mode of migration to mesenchymal cell migration but retains cell-cell contacts with neighbouring cells. Such collective cell migration can be seen in bulk cancer cell migration and migrating epithelial cells during wound healing [1, 2]. Switching between collective and single cell migration is an important regulator of cancer metastasis and is often associated with altered gene expression.

**Figure 1:**
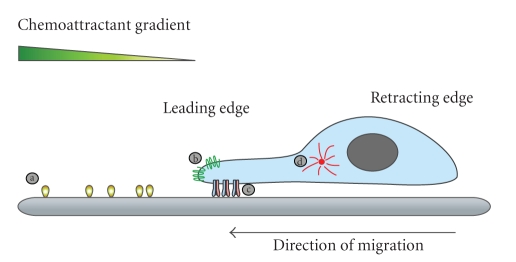
Cell migration. Responding to extracellular cues such as chemoattractant gradients or adherent cues (a), the migrating cell changes its morphology and intracellular organization. Polarised cells have a fan-shaped protrusion at the leading edge and traction at the rear. Chemoattractant receptors (b) and integrins (c), forming focal adhesions, localise to the leading edge of the cell. The microtubule organising centre (MTOC) (d) and the Golgi locate to the side of the nucleus that faces the leading edge.

There are general similarities in single cell migration across cell types, although different cell types have unique features and characteristics. Here, we briefly summarize the general stages of migration since they are frequently used to describe and classify migratory behaviour in cell studies with engineered environments. Cell migration can be divided into four phases: polarisation, protrusion, traction, and disassembly (Figure 1). 

In response to extracellular cues, the cell polarises where specific proteins and lipids accumulate asymmetrically in the anterior and posterior of the cell. During the polarization phase, proteins, lipids and organelles are rearranged in the opposite poles in the cell in order to perform their specific functions in cell migration. Often recruited by ligand-receptor interaction, markers for the leading edge in mesenchymal migration are phosphoinositide 3-kinases (PI3K) [3], the gangliosides GM1 and GM3 [4], phospholipase D [5] while tensin homolog deleted on chromosome 10 (PTEN) is located at the rear [6]. In amoeboid migration, GM3 is also located at the leading edge but GM1 locates to the rear [4]. Rearrangement of organelles also contributes to persistent polarisation. The microtubule organising centre (MTOC) and the Golgi are typically located on the side of the nucleus that faces the leading part of migrating cells [7].

Most polarised cells have a ruffled, fan-shaped protrusion at the leading edge and traction at the rear. At this stage, the cell establishes firm connection with the surrounding matrix or substratum in the form of a nascent adhesion spot, called focal complex and mature adhesion sites called focal adhesion. Attachment of actin stress fibres to focal adhesions, in conjunction with molecular motors, generates forces that move the cell body forward. Finally, a new adhesion forms at the leading edge and old focal adhesions at the rear are disassembled allowing the cell to advance.

## 2. Soluble Cues and Directed Cell Migration

Cell signalling molecules such as growth factors, hormones, chemokines, and microbial epitopes can trigger cell migration. These ligands can activate transmembrane receptors such as receptor tyrosine kinases, cytokine receptors and G protein coupled receptors. Triggering of these cell surface receptors recruits signalling proteins to the activation site and consequently intracellular signalling cascades. If the ligands for these receptors are spatially encoded, these processes initiate cell polarization and define the direction of migration towards higher ligand densities.

The classic chamber to measure directed cell migration, or chemotaxis, is the transwell Boyden chamber. With the transwell Boyden chamber cells are plated on a porous membrane and the chemoattractant is placed in a chamber beneath the membrane. Hence, cells are attracted to migrate through the porous membrane. Unfortunately, these chambers do not allow the visual inspection of the locomotion and are typically used to quantify the percentage of migrating cells. Various microscope setups [8] for directed cell migration have been designed; for example, micropipettes can be used as a point source for chemoattractants or an agarose gel in the so called “under-agarose” assay, in which cells and chemoattractant in solution, are placed inside wells cast into an agarose layer [9]. In all of these designs, the chemoattractant can freely diffuse in solution, which makes it difficult to accurately delivery a known concentration to the migrating cell.

**Figure 2:**
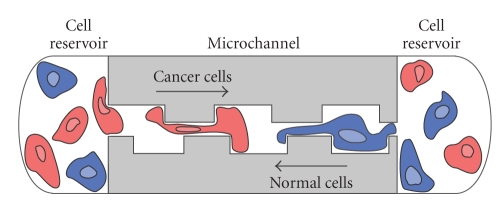
Directed migration through microchannels engineered with soft lithography techniques. Cancerous cells (red) show different morphology and migratory behaviour than non-cancerous cells (blue) when cocultured in a microchannels of a particular geometry resulted in directed migration [15]. Soft lithography can be used to engineer the microchannels in rigid or soft materials.

Recently, microfluidic applications and soft lithography technique have been employed to delivery soluble cues to migrating cells [10, 11]. In microfluidics, the fluid flow is controlled by viscous force (laminar flow) and in some cases, cells or proteins placement is controlled by electrical stimulation [12, 13]. Microfluidics channels are traditionally made of silicon, glass and other rigid materials but more recently elastomeric polymers such as poly(dimethylsiloxane) (PDMS) have begun to be extensively used. To engineer the microfluidics channels (Figure 2) microcontact printing, which is part of the suite of soft lithography methods but no longer requires soft stamps, is particularly useful because it transfers patterns by stamping and is therefore a nonphotolithographic technique [14].

Because microfluidic channels can be designed in various sized, this approach is suitable for study of single cell migration [15] and cell motility in bulk [11]. Various types of fluidic and fluid-driven mechanical stimuli can be applied for creating gradients, in the microfluidic channel, of growth factors [16], chemoattractants and chemorepellents [10, 17], or drugs [18]. In addition, modifications of microfluidics design can be used to study the effect of channel topography, surface pattern, and surface dimensions on cell migration. For instance, the biased migration response of two different cell types was recently reported using microratchet channels. Interestingly, cancerous cells showed different morphology during migration than noncancerous cells expanding their protrusion pass the boundary of trapezium opening while non-cancerous fibroblast lamellipodia do not broaden its lamellipodia to the trapezium border but anchor to the nearby spike on the trapezium opening [15] (Figure 2).

Other variables can be controlled using microfluidics devices such as adhesive cues [19], shear stress [20], and oxygen level [21]. Microchannels were recently used to test how migration speed is determined by the deformability of cancer cells [22]. This study also highlighted the different motions of cells in confined 3D environment compared to migration over flat surfaces [22].

## 3. Cell Adhesion and Integrins

**Figure 3:**
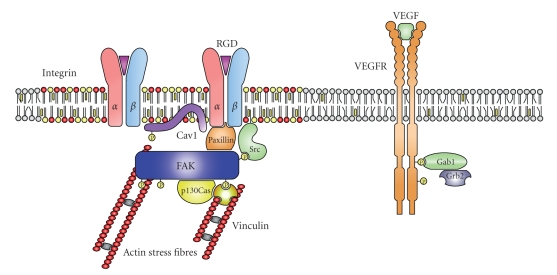
Integrin and VEGF-receptor signalling. Focal adhesions (FAs) are large complexes, which consist of integrins and VEGF-receptors (VEGFR). The *α*- and *β*-subunit of integrins bind to the tripeptide arginine-glycine-aspartic acid (RGD) in fibronectin and other proteins of the extracellular matrix (ECM). This induces a signalling cascade that ultimately leads to restructuring of the actin skeleton and cell migration. Activation of integrins induces phosphorylation of focal adhesion kinase (FAK) by the receptor tyrosine kinase Src. Paxillin is a focaladhesion associated adaptor protein. It interacts with several other focal adhesion proteins such as talin, tensin and vinculin. P130Cas is an adaptor protein that induces signalling cascades involving ERK. VEGFR translocates to focal adhesions after stimulation with VEGF and initiates a signalling cascade, which contributes to focal adhesion organization and actin restructuring.

Focal adhesions are the sites of cell connection to ECM where the cell's actin cytoskeleton is tethered to the ECM's nanofibres. The physical link between the outside and inside of the cell is achieved by transmembrane proteins, mainly integrins. Integrins are members of an *α*/*β*  heterodimeric receptor family [23] (Figure 3). In mammals, there are 18 identified *α* subunits and 8*β* subunits that can combine to give 24 distinct heterodimers. Many integrins are expressed in a low-affinity binding state. However, cells can change integrin conformation and hence affinity in response to cellular stimulation, a process termed integrin activation. The signals leading to integrin activation are referred to as “inside-out signalling”. Integrin activation results in renewed probing of the cell's environment and, when activated integrins become engaged to their ligands, focal adhesion formation. Thus, focal adhesions contain a high concentration of activated and engaged integrins. In response to growth factors, many cells alter both the repertoire and affinity of integrins.

In addition to the inside-out signalling, focal adhesions control a range of cell activation responses, such as cell polarization and migration, membrane trafficking, cell cycle progression, gene expression, and oncogenic transformation [24, 25]. Focal adhesions are large protein complexes, which initiate “outside-in signalling” involving the phosphorylation of focal adhesion kinase (FAK), p130 Cas, Src and other tyrosine kinases, phosphorylation of the structural membrane protein caveolin-1 (Cav1), phosphoinositide (PI) 3-kinases, the kinases Erk, JNK, and p38 MAP kinases, as well as small GTPases [26, 27] (Figure 3). In addition, actin-regulating proteins such as vinculin, paxillin and talin link integrin complexes to actin stress fibres. Focal adhesions are also connected to growth factors signalling. For example, vascular endothelial growth factor (VEGF) stimulation of endothelial cells results in the translocation of its type 2 receptors (VEGFR2, KDR/Flk-1) from caveolae to focal adhesions. Dimerization of the receptors initiates autophosphorylation but it is at FA where VEGF receptors induce signalling cascades [28]. In return, VEFGR2 also activates integrins *α* *v* *β*3, *α*5*β*1, and *α*2*β*1 [29], and the Rho GTPase Rac1 bridging signalling activities with focal adhesion organization and actin restructuring [30–32]. 

Curiously, integrins have no intrinsic enzymatic activity yet in many cases, integrins enable growth factor signals—that is, growth factor signalling does not occur unless integrins are occupied. Continuing with the example of endothelial cells, *α* *v* *β*3 antagonists can inhibit angiogenesis [33], indicating the importance of these integrins in angiogenesis [34]. However, integrins can also suppress growth factor signalling. Genetic deletion of *α* *v* integrins has only modest effects on angiogenesis [35] while genetically deleting integrins *β*3 and *β*5 enhances normal and pathological angiogenesis [36]. Transdominant integrin inhibition, a form of integrin crosstalk, may account for some of the effect of *α* *v* *β*3 ligands and antagonists on angiogenesis. There is now considerable evidence that physical and topographical characteristics of integrin ligands regulate integrin function (reviewed recently in [37]).

## 4. Model Substrata to Study Cell Attachment and Migration

Because of the crucial role of focal adhesions in both cell adhesion and signal transduction, extensive research has been undertaken to understand the molecular architecture of adhesion sites. Fluorescence microscopy studies are the methods of choice because they enable insights into intact, live cells.

To identify the molecular architecture of focal adhesions, one needs to create surfaces on which the interactions with integrins can be precisely controlled and which mimic the arrangement of adhesive sites in the ECM. Coating glass or plastic surfaces with ECM proteins was the starting point used to identify integrin specificities but because neither the amount nor the location of the ECM protein can be controlled, molecular mechanisms are difficult to identify with this approach. A characteristic feature of the ECM is the periodic nature of integrin ligands. Native collagen, for example, has a fibril structure with each fibril displaying a periodicity of ~67 nm [38]. This periodicity can increase to 250 nm fibrous collagen [39]. Similarly to collagen, fibronectin is organized into nanoscale patterns, exhibiting paired fibrils with characteristic spacings of 156, 233, 304, and 373 nm [40] while integrin binding sites with fibronection fibre, the tripeptide arginine-glycine-aspartic acid or RGD, have a periodicity below 70 nm. Hence, it is desirable to engineer surfaces on which integrin ligands have precise intermolecular distances of <5 nm to >500 nm. 

Adsorbing matrix proteins and modifying polymers provide insufficient control over ligand presentation while microcontact printing and dip-pen lithography achieve at best patterns of 100 nm separation. The first attempt to control the presentation of RGD ligands at the surface was to covalently graft RGD peptides onto a polymer substrate [41, 42]. Using these surfaces it was found that cell-substrate interactions depended on RGD density. However, in this approach, RGD peptides were attached to the heterogeneous environment of the polymer gel. As a consequence, the number of RGD peptides accessible by cells differed from the total of RGD within the gel, making it impossible to precisely determine the effective density. Later, this strategy was refined to probe for the optimal RGD spacing for cell adhesion and spreading using glass substrates modified with a polyethylene oxide (PEO) polymer to which RGD peptides were grafted [43] and star-shaped PEO molecules controlling the number of RGD peptides per macromolecule [44]. These studies revealed RGD spacing of 440 nm was sufficient for fibroblast spreading but focal adhesion formation required higher RGD densities [43] or a clustered ligand arrangement [44].

Microcontact printing [45], combined with self-assembled monolayer chemistry [46], and block copolymer nanolithography [47, 48] can space integrin ligands closer than 200 nm apart. When spacing integrin ligands that preciously, one has to ensure that the surface between the ligands is sufficiently passivated so that cells specifically interact with the presented ligands and not with the underlying surface. Oligo(ethylene oxide) been shown to be effective in preventing protein absorption [49] and cell adhesion [50] and gold surfaces modified with poly(ethylene glycol)-terminated alkanethiol monolayers [49] ensure specific interactions between the integrins and RGD ligands. Such surface modification strategies demonstrated the importance of density [43, 44, 51], affinity [52], and spatial organization [44, 53] of RGD ligands on cell adhesion and spreading. However, because gold surfaces quench fluorescence, these studies were limited to morphological descriptions. 

Spatz and coworkers used block copolymer nanolithography (BCN) to position gold nanodots with high precision at 28, 58, 73, 85, and 108 nm spacing with each nanodot engaging only a single integrin [54, 55]. The analysis of focal adhesion dynamics on homogeneously spaced [47], disordered [56] or gradients [48] of cyclic RGD peptide revealed that RGD-to-RGD spacing that exceed >70 nm result in less cell spreading, higher focal adhesion turnover and more erratic cycles of membrane protrusion and retraction in fibroblasts. This detailed analysis of focal adhesion proteins and turnover suggested that there is an optimal RGD density for integrin engagement. Importantly, the 10–200 nm scale of ligand spacing is physiologically relevant as nanoscaled and periodic spacing of integrin ligands below 100 nm is found in fibronectin and collagen fibers [57, 58]. In conclusion, cells have the remarkable ability to sense variation in spacing of integrin ligands on the nanometre scale [37].

In contrast to 2D surfaces, our understanding of the effect of surface topography on cell adhesion and migration is limited although substrate topography could affect the ability of cells to orient and migrate as well as influencing the organization of their cytoskeleton [59]. It was found that on striated substrates, endothelial cells, smooth muscle cells, and fibroblasts align and migrate along grooves and that cell orientation is increased with smaller lateral spacing and increasing depth of the grooves present at the surface [60]. The choice of substrate and the chemical patterns on surfaces [61] may also be important. This issue has mainly been addressed by varying the chemical composition of the substrate itself using different metal alloys [62] or polymers [63] but these substrata were devoid of integrin ligands. In contrast, coating titanium with RGD proved to stimulate bone cell adhesion, but the interfaces lacked control over peptide surface density [64]. Particularly for cancer research, model substrata with controllable flexibility, topography, chemical modifications, and nano-scaled patterning would provide a better understanding of the impact of the cell's microenvironment on cell migration.

## 5. Conclusion

Recent advances in microfluidics, nanopatterning and surface chemistry have afforded unique insights into how cells sense and respond to their local environment [37]. Microfabrication enabled the engineering of microfluidic channels of various topography and shown that the direction of migration is determined by protrusion morphology [15]. Cell adhesion onto 2-dimensional surfaces revealed that cells adhere and spread differently if the density [47], patterning [48], or degree of ordering [56] of the integrin ligands is altered on the nanometre to micrometer scale [65] influencing the distribution of focal adhesion proteins [47] and structuring of the actin cytoskeleton [66]. Only by integrating these advance to, for example, examine cell migration over previously engineered 2D surface in microfluidics devices, or modifying the surface chemistry of microfluidics channels can we understand how cells integrate soluble and adhesive cues in their decision making processes. The fundamental insights into cell adhesion and migration will add the engineering of appropriate cellular environments in medical implants [65], biosensors [67], and tissue materials [68].
